# Population pharmacokinetics of levodopa gel infusion in Parkinson’s disease: effects of entacapone infusion and genetic polymorphism

**DOI:** 10.1038/s41598-020-75052-2

**Published:** 2020-10-22

**Authors:** M. Senek, D. Nyholm, E. I. Nielsen

**Affiliations:** 1 Department of Neuroscience, Neurology, Uppsala University, Uppsala, Sweden; 2 Department of Pharmacy, Uppsala University, Uppsala, Sweden

**Keywords:** Movement disorders, Parkinson's disease, Medical genetics

## Abstract

Levodopa-entacapone-carbidopa intestinal gel (LECIG) provides continuous drug delivery through intrajejunal infusion. The aim of this study was to characterize the population pharmacokinetics of levodopa following LECIG and levodopa-carbidopa intestinal gel (LCIG) infusion to investigate suitable translation of dose from LCIG to LECIG treatment, and the impact of common variations in the dopa-decarboxylase (DDC) and catechol-O-methyltransferase (COMT) genes on levodopa pharmacokinetics. A non-linear mixed-effects model of levodopa pharmacokinetics was developed using plasma concentration data from a double-blind, cross-over study of LCIG compared with LECIG in patients with advanced Parkinson’s disease (n = 11). All patients were genotyped for rs4680 (polymorphism of the COMT gene), rs921451 and rs3837091 (polymorphisms of the DDC gene). The final model was a one compartment model with a high fixed absorption rate constant, and a first order elimination, with estimated apparent clearances (CL/F), of 27.9 L/h/70 kg for LCIG versus 17.5 L/h/70 kg for LECIG, and apparent volume of distribution of 74.4 L/70 kg. Our results thus suggest that the continuous maintenance dose of LECIG, on a population level, should be decreased by approximately 35%, to achieve similar drug exposure as with LCIG. An effect from entacapone was identified on all individuals, regardless of COMT rs4680 genotype. The individuals with higher DDC and COMT enzyme activity showed tendencies towards higher levodopa CL/F. The simultaneous administration of entacapone to LCIG administration results in a 36.5% lower apparent levodopa clearance, and there is a need for lower continuous maintenance doses, regardless of patients’ COMT genotype.

## Introduction

Levodopa/carbidopa intestinal gel (LCIG) is a treatment developed for patients with advanced Parkinson’s disease (PD) when oral treatment fails to provide sufficient stability in symptom relief^1^. Continuous infusion of drug, resulting in a more stable plasma concentration, stabilizes the symptom fluctuations (on–off phenomenon) as well as decreases the time with dyskinesia (levodopa-related involuntary movements)^2^. The levodopa/entacapone/carbidopa intestinal gel (LECIG) is a gel with the addition of entacapone^3^. Entacapone is a reversible inhibitor of catechol-O-methyltransferase (COMT), the enzyme responsible for the second major metabolic pathway of levodopa. The addition of carbidopa causes inhibition of dopa decarboxylase (DDC), which is the enzyme responsible for the largest part of levodopa’s metabolism. The addition of entacapone has shown to allow lower levodopa dose administration through the inhibition of COMT, thus increasing levodopa plasma concentrations^3^.

The drug-containing gel is infused directly into the small intestine, via a gastrojejunostomy tube, bypassing the stomach, and is thereby not affected by gastric emptying, which usually has a negative and erratic impact on levodopa absorption. The infusion treatment, most commonly administered only during day-time, consists of a morning bolus dose and a continuous maintenance infusion. The morning bolus dose is administered at the highest pump rate (40 mL/h) to allow levodopa to rapidly reach therapeutic plasma concentrations. When initializing LCIG treatment, the doses are based on the patients’ previous oral levodopa morning dose, and total daily dose. Patients can also administer small bolus doses (extra doses) during the day, if needed.

A previous pilot study was conducted where oral entacapone (200 mg, every 5 h) was added to LCIG treatment. With a 20% decrease in LCIG dose with the COMT inhibitor, the plasma concentrations at steady state (0.5–8 h) did not differ compared to LCIG administered alone without dose adjustment^4^. In a clinical trial investigating the infusion of LECIG using a 20% reduction of morning and maintenance infusion doses, the morning levodopa plasma concentrations were found to be lower than following infusion of LCIG, and there was a trend towards an accumulation in levodopa concentrations throughout the day^3^. This may result in insufficient symptom relief in the morning and an increased risk of dyskinesia in the latter part of the day. The LCIG treatment was highly individualized, with morning and continuous maintenance doses to meet individual patient needs. The patients were also allowed to administer extra doses if needed, which complicates the data analysis when using conventional area-based methods.

It was also observed that not all patients had the same increase in levodopa plasma concentration with the new treatment^3^, and it was hypothesized that a reason for this could be differences in enzyme activity. Genetic variations in the gene encoding for the enzyme COMT (rs4680), and in the DDC promoter gene (rs921451 and rs3837091) have been suggested to affect the natural activity and/or expression of the respective enzymes, which in turn may affect the pharmacokinetics of levodopa^5,6^. Hypothetically, an individual with e.g. high COMT activity may benefit the more from the addition of entacapone, and polymorphism related to DDC might be correlated to the effect of carbidopa on levodopa pharmacokinetics.

The aim of this analysis was to investigate the impact of simultaneous entacapone infusion on levodopa pharmacokinetics using a model-based approach to provide a translation of dose from LCIG to LECIG treatment, based on previously published data^3^, and to investigate the effect on levodopa pharmacokinetics by genotypes of the DDC and COMT genes.

## Methods

### Study population

Eleven patients were included in a randomized, open-label, 2-day crossover clinical trial (Table 1)^3^. The local Ethical Review Board in Uppsala, Sweden and the Swedish Medical Products Agency approved the study, and all patients provided written informed consent. All research was performed in accordance with relevant guidelines/regulations. For two consecutive days, patients were randomized to receive one of two treatment sequences, LECIG/LCIG or LCIG/LECIG. LECIG morning doses corresponded to 80% (n = 5) or 90% (n = 6) of their LCIG morning dose, 80% of the LCIG continuous maintenance dose, and 80% of the dose for extra bolus administrations. The treatment duration was 14 h, at which point the tube was immediately flushed with water, as is required with both treatments. When flushed, the gel left in the tube, approximately 3 mL, is infused. This volume corresponds to 60/15 mg of levodopa/carbidopa (LCIG) and 60/60/15 mg of levodopa/entacapone/carbidopa (LECIG). Oral levodopa-carbidopa immediate-release tablets were allowed as night-time medication after infusion stop and until 3 h before the infusion start. During the study, low-protein meals were served at hour 1, 4, 7, 10 and 13 after infusion start. The mean (min, max) of protein in grams at each time point was 8.8 (5.7,12), 10.8 (9.3,12), 2.1 (2.0,2.3), 10.3 (8.9,11), 5.4 (3.0, 6.3) day 1 and 8.8 (5.7,12), 10.8 (9.3,12), 2.1 (2.0,2.3), 9.9 (5.8,11), 5.3 (3.0, 6.0) day 2.

**Table 1:** Patient characteristics, n = 11 (male n = 7, female n = 4).

	Age (years)	Duration PD (years)	Duration LCIG (years)	Body weight (kg)	LCIG formulation		LECIG formulation	
					Morning dose (mg) n = 10^a^	Maintenance dose (mg)	Morning dose (mg) n = 10^a^	Maintenance dose (mg)
Mean (SD)	70 (4)	16 (4.8)	2.7 (2.7)	74 (15)	131 (56)	969 (277)	120 (49)	772 (226)
Median	70	14	1.3	73	130	1048	122	824
Min, max	63, 76	8, 23	0.2, 7.6	51, 99	41, 217	363, 1367	41, 198	279, 1107

^a^One patient did not have a morning dose prescribed. SD, standard deviation; PD, Parkinson’s disease; LCIG, levodopa-carbidopa intestinal gel; LECIG, levodopa-entacapone-carbidopa intestinal gel.

Blood samples were drawn immediately prior to dosing, half-hourly between 0 and 3 h, and hourly between 3 and 14 h. A blood sample was collected within 5 min after flushing and then half-hourly between 14.5 and 17 h.

### Sequence variations of DDC and COMT genes

All patients that were included in the study submitted a blood sample for genotyping of DDC and COMT polymorphism, after providing written informed consent. Genomic DNA was extracted from the blood samples and the single nucleotide polymorphisms (SNP) rs4680 (COMT_SNP_) and rs921451 (DDC_SNP_) were analyzed by allelic discrimination TaqMan assay. The SNP in the COMT gene (rs4680)^5,7^ results in the substitution of A > G, which causes the conversion of the enzyme valine (158Val, higher activity) to methionine (158Met, lower activity). The 158Val allele is associated with a higher enzymatic activity of COMT. The SNP in the DDC gene (rs921451)^6,8^ results in a nucleotide substitution of T > C, which is associated with lower expression and/or activity. For identification of the DDC gene (rs3837091) polymorphism (DDC_INSDEL_), the Sanger sequencing method was used, and the amplicons were compared to a GenBank-reference sequence. The polymorphism (rs3837091)^7^ is characterized by a 4-base pair deletion (AGAG), which may cause lower expression and/or activity of DDC. For each patient, one control for each genotype was analyzed. Any difference in CL/F for the DDC_SNP_, DDC_INSDEL_ and COMT_SNP_ were graphically explored, based on empirical Bayes estimates.

### Model development

#### Base model

Initially, a population pharmacokinetic model was developed with shared parameters for both treatments. Thereafter, differences in parameter estimates were successively investigated, to evaluate the impact of simultaneous entacapone infusion. One and two compartment disposition models with first order absorption were evaluated, parameterized in terms of absorption rate (ka), relative bioavailability (Frel), apparent volume of central (V_C_/F) and peripheral (V_P_/F) compartment, apparent clearance (CL/F) and inter-compartmental clearance (Q/F). Inter-individual variability was included assuming a log-normal distribution of structural model parameters. Bodyweight was included as a primary covariate on all disposition parameters according to the allometric power model, with allometric power exponents or 0.75 for CL/F and 1 for V/F^9^. Oral levodopa-carbidopa tablets were allowed as night time medication during the study, but only until 3 h before morning dose. Eight individuals took night-time medication 01:10–05:50 h after stop of LECIG administration and 01:43–04:28 h after stop of LCIG, and very few blood samples were collected in relation to the oral treatment. Thus, the information available was too sparse to allow for estimation of the absorption related parameters for the oral levodopa treatment. Therefore, based on a previously published levodopa pharmacokinetic model where oral levodopa-carbidopa tablet administration was compared to LCIG^10^, the absorption model for oral treatment was described with a single transfer rate constant fixed to 2.4 h^-1^ with one transit compartment between the depot and central compartment, and a relative difference in Frel of 1.03. Since number of levodopa measurements below the limit of quantification was low (1.9%) these samples were handled using the M6 method^11^, where LOQ/2 is assigned to the first value and subsequent samples below LOQ were ignored. The difference in levodopa parameters for LECIG were investigated as a relative difference in the estimate of CL/F, ka and F_rel_ compared to LCIG. The effect of food intake was explored both as a binary variable (yes/no), and as a continuous variable reflecting the amount of protein intake, that was assumed to decrease the drug absorption during an estimated period following food intake. For investigation of dosing regimens**,** a simulation dataset was created with the same number of individuals and the same demographic characteristics as the individuals included in the model development dataset. The model was used to simulate 1000 datasets, where individuals were dosed with either the same or altered dose regimens.

#### Data analysis and model evaluation

The population pharmacokinetic model was developed using the non-linear mixed effects modelling software NONMEM^12^ (version 7.3; Icon Development Solutions, Ellicott City, MD, USA, 2009) with the first order conditional estimation method with INTERACTION (FOCEI) and a user-defined model (ADVAN13 NONMEM Subroutine). PsN^13^ (version 4.7.0; Department of Pharmaceutical Biosciences, Uppsala University) was used for running models.

Parameter precision, scientific plausibility, goodness-of fit plots, prediction corrected visual predictive checks (pcVPCs)^14^, and the objective function value (OFV) were used for model evaluation during the model development process. The OFV (approximates − 2 log(likelihood) of the data given the model) was utilized in likelihood ratio testing (LRT) to compare nested models (a ∆OFV of 3.84 for 1 degree of freedom, corresponding to a significance level of 0.05 was used). R^15^ (version 3.4.2; R Foundation for Statistical Computing) was used for data management and Xpose^13^ (version 4.6.0; Department of Pharmaceutical Biosciences, Uppsala University) was used for graphical evaluation. Parameter uncertainty of model parameters was assessed with the Sampling Importance Resampling (SIR) procedure^16^. The adequacy of the final model was evaluated using pcVPCs with 1000 replicates of the observed data.

### Ethics approval

The local Ethics Review Board in Uppsala, Sweden and the Swedish Medical Products Agency approved the clinical trial. The genotyping part of the study was separately approved by the Ethics Review Board. All patients provided written informed consent.

## Results

The final population pharmacokinetic model was a one-compartment model parameterized in terms of ka, Frel, Vc/F and CL/F. The estimated absorption rate constant (ka) for both treatments was very high, and was therefore fixed to 50 h^-1^, corresponding to the lowest value which did not give a significant increase in OFV. Estimation of a two compartment model resulted in an OFV drop of 23, however the distribution phase was estimated to be very fast and the model became unstable with high uncertainty on the estimated parameters. Inter-individual variability was explored on all parameters, and found to be significant on CL/F and Vc/F. Addition of an inter-individual variability on relative bioavailability resulted an OFV drop of 5.85, but was associated with a high relative standard error (190%) and model instability, and was therefore not retained in the model. The model improved, with a difference in OFV of − 436, when the effect of entacapone was estimated as a shift in the typical value of levodopa CL/F, including an inter-individual variability in the shift parameter. The population parameter for CL/F was estimated to 27.9 L/h/70 kg for LCIG and to be 36.5% lower for LECIG, with associated inter-individual variabilities of 28% and 11%, respectively. All final model parameter estimates are given in Table 2.

**Table 2:** Parameter estimates for the final population pharmacokinetic model of LCIG and LECIG, and results from the SIR evaluation.

Parameter	Point estimates (%RSE)^b^ [% Shrinkage]	SIR (%RSE)^b^ [95% CI]
CL/F_LCIG_ (L/h/70 kg)	27.9 (7.31)	28.1 (5.82) [25.1; 31.5]
CL/F_LECIG,Shift_^a^	− 0.365 (5.24)	− 0.364 (4.48) [-0.391; − 0.328]
V_C_/F (L/70 kg)	74.5 (7.60)	75.0 (8.60) [63.3; 87.8]
ka (h^−1^)	50 FIX	–
ktr_oral_ (h^−1^)	2.4 FIX	–
F_rel,LCIG/LECIG_	1 FIX	–
F_rel,oral_	1.03 FIX	–
IIV_CL/F,LCIG_	27.9 (19.8) [1E−10]	28.6 (14.8) [21.2; 36.2]
IIV_CL/F,LECIG,Shift_^a^	11.4 (23.5) [22.6]	12.0 (30.1) [4.49; 17.9]
IIV_VC_	34.4 (17.0) [0.264]	35.6 (17.2) [24.2; 45.7]
Proportional error (%)	11.0 (27.4)	11.1 (8.96) [3.24; 13.1]
Addititive error (µg/mL)	0.316 (10.2)	0.316 (6.14) [0.278; 0.354]

^a^Shift in CL/F for LECIG, $CL/F_{i} = TVCL/F_{LCIG} \times e^{CL,LCIG} \times \left( {\frac{Weight}{{70}}} \right)^{0.75} \times \left( {1 + TVCL_{LECIG,Shift} \times e^{CL,LECIG,Shift} } \right)$.

^b^NONMEM point estimate and the associated % relative standard error (% RSE, reported on the approximate standard deviation scale (SE/variance estimate)/2). CI, confidence interval; IIV, inter-individual variability (CV%). SIR, sampling importance resampling.

**Figure 1:**
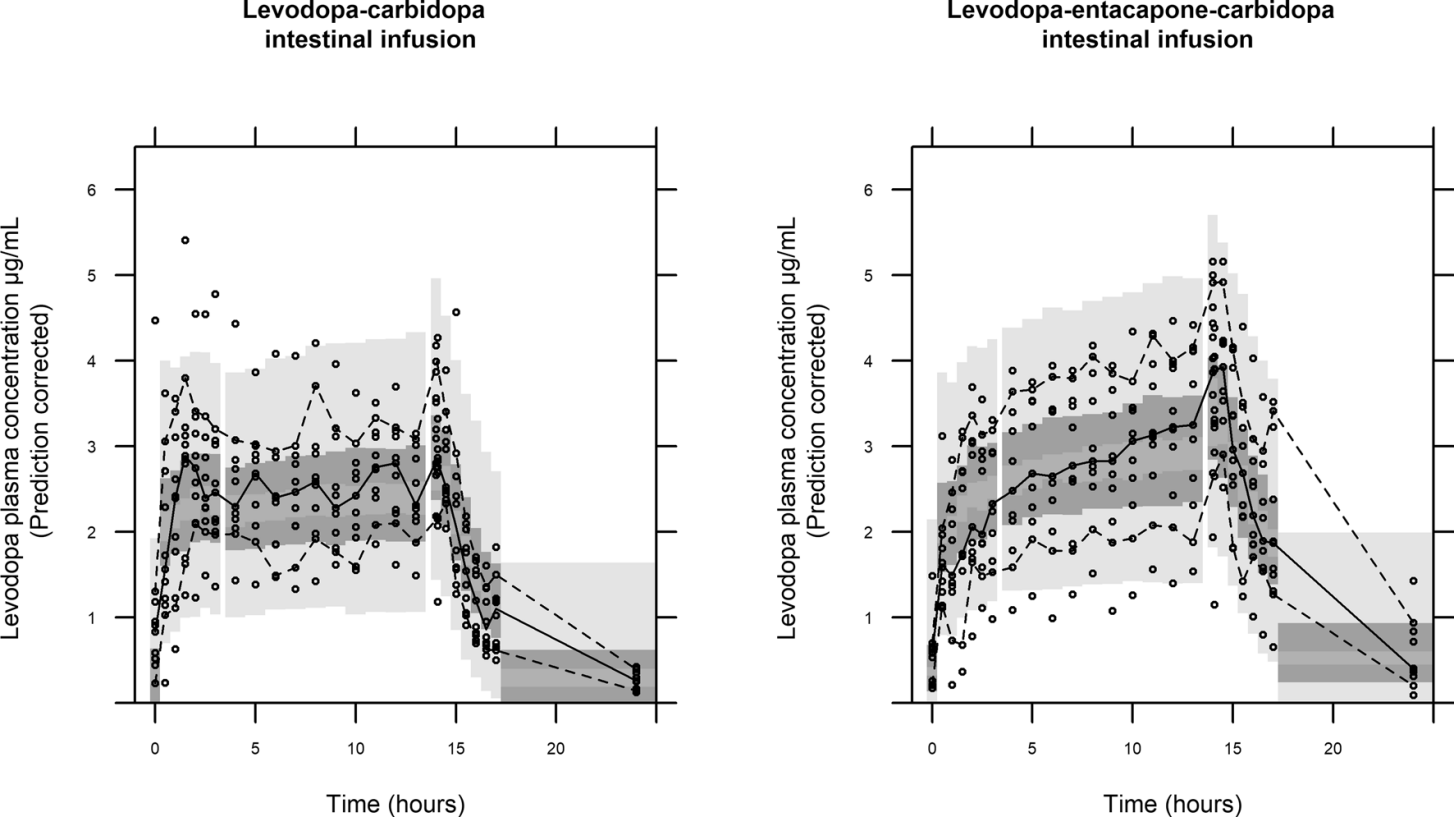
Prediction corrected visual predictive check (1000 samples) of the concentration–time data for LCIG and LECIG. The solid line is the median of the observed data. The dashed lines represent the observed 10th and 90th percentiles of the observations. The top and bottom light grey areas are the 95% confidence intervals for 10th and 90th percentiles of the simulated data. The middle dark grey area is the 95% confidence interval for the median of the simulated data.

The pcVPC, showing the observed and model predicted levodopa plasma concentration normalized for the variability in the independent variables, stratified on treatment is shown in Fig. 1. The observed plasma concentrations are in general well predicted by the model for both treatments.

**Figure 2:**
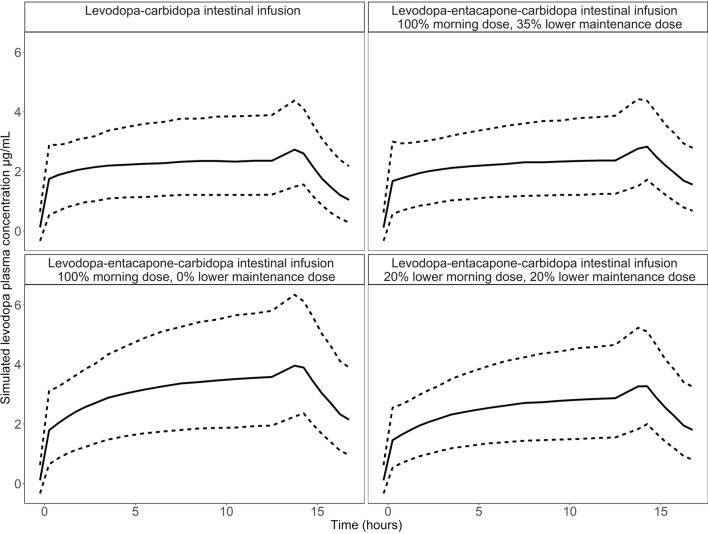
Simulated plasma concentration for the study population, with unchanged patient doses of LCIG as reference (top left plot) and decreased continuous doses for LECIG treatment by: 0% lower morning dose and 35% lower maintenance dose (top right plot), 0% lower morning and maintenance dose (bottom left plot) and 20% lower morning and maintenance dose (bottom right plot). The solid line represents the median of the simulated data, the top and bottom dashed lines represent the 10th and 90th percentiles of the simulated data.

The developed population levodopa pharmacokinetic model, which describes the time course of drug exposure in patients, was used to simulate alternative dose regimens for LECIG. In the scenarios, both morning bolus dose and continuous maintenance dose were altered. The infusion period simulated was 14 h. The scenarios included no dose adjustment (i.e. 0% lower morning dose and maintenance dose); 20% lower morning and maintenance dose and; 0% lower morning dose with a 35% lower continuous maintenance dose, compared to LCIG. Figure 2 shows a comparison of the levodopa plasma concentration of the three LECIG scenarios compared to LCIG administration. The levodopa plasma concentration is displayed as the median and the 10th and 90th percentiles. Administration of the same levodopa dose with LECIG as with LCIG, i.e. 0% lower morning and continuous maintenance dose, shows that the predicted plasma concentration increases during the infusion period. In the original study, a 20% lower morning dose and maintenance dose was given, and as previously observed, this results in a slight increase in levodopa plasma concentrations over the 14-h infusion period. A decrease of the continuous maintenance dose by 35% results in similar drug exposure as LCIG, indicating that, on a population level, this would be an appropriate dose adjustment.

**Figure 3:**
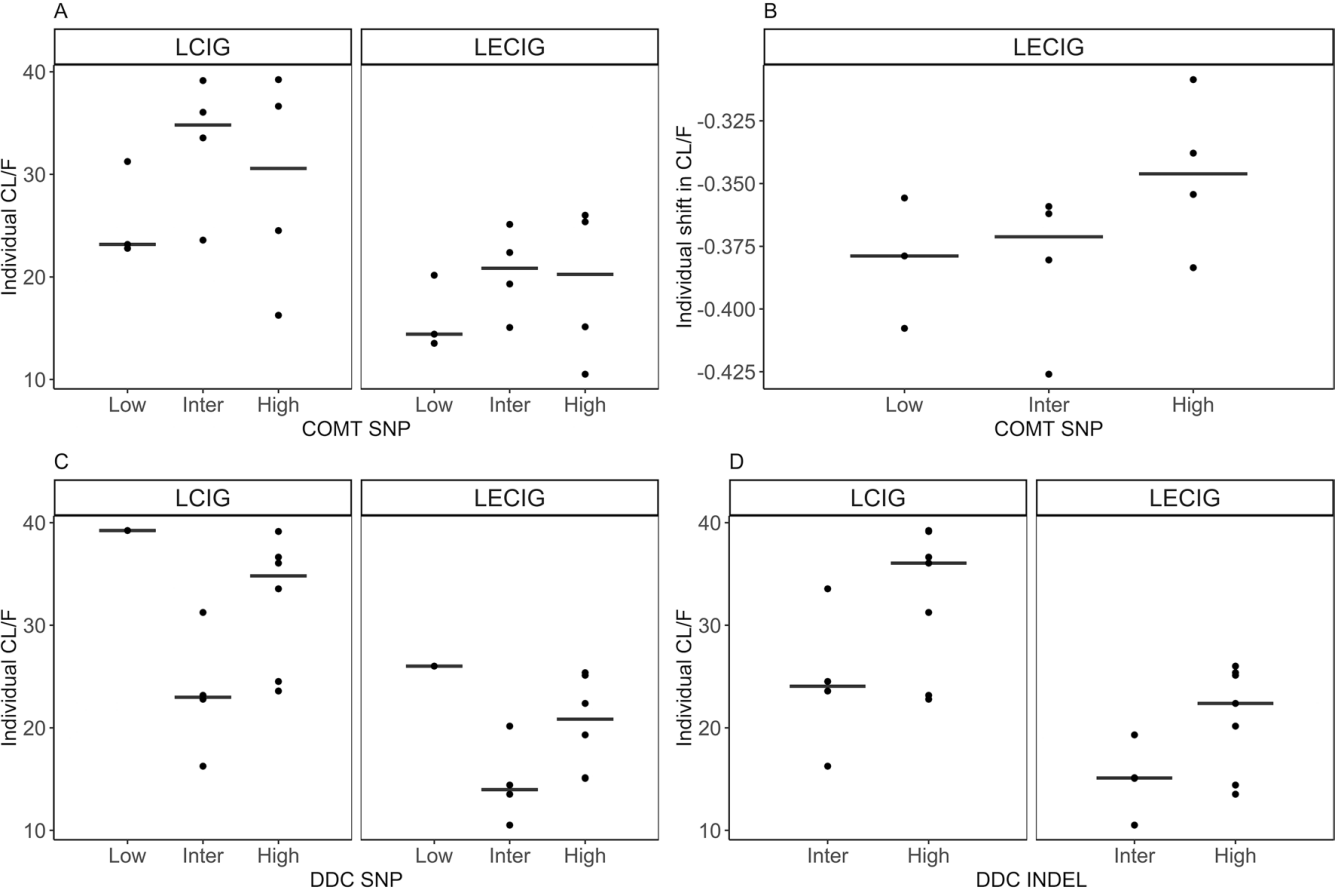
Graphical analysis of genotype results and individual estimated LD CL/F, with LCIG and LECIG treatment. COMT (rs4680, top left, A) and individual shift CL/F (top right, B), DDC_SNP_ (rs321451, bottom left, C), DDC_INSDEL_ (rs3837091, bottom right, D). The middle line represents the median of the data.

The results of estimated individual CL/F, for levodopa with and without entacapone, stratified on genotype are shown in Fig. 3, together with a plot showing the individual shift in CL/F with the addition of entacapone. The results from the COMT_SNP_ (rs4680) genotyping showed that three patients had genotype COMT^AA^ (low), four patients had COMT^AG^ (intermediate) and four patients had COMT^GG^ (high). There is no clear trend observed in CL/F between the COMT activity subgroups (Fig. 3A). All COMT_SNP_ genotypes display a decrease in CL/F with the addition of entacapone (Fig. 3B). One patient had been genotyped with DDC_SNP_^CC^ (low), four patients with DDC_SNP_^CT^ (intermediate) and six patients with DDC_SNP_^TT^ (high activity). Patients with high activity of DDC, based on the single nucleotide polymorphism results, showed a tendency to have a higher CL/F (Fig. 3C). However, the one patient with a low activity, had an estimated CL/F that was higher compared with the other two groups. This patient, on the other hand, has a high activity of DDC based on the DDC_INSDEL_ and of COMT according to COMT_SNP_. The DDC_INSDEL_ genotyping (rs3837091), revealed four patients with DDC_INSDEL_^AGAG/–^ (intermediate), and seven patients with DDC_INSDEL_^AGAG/AGAG^ (high). Patients with intermediate activity seem to have slightly lower median CL/F, compared with patients with high activity (Fig. 3D).

## Discussion

In this analysis, the difference in levodopa pharmacokinetics, administered as an intestinal infusion with and without simultaneous entacapone infusion was investigated using a population modelling approach. Following oral administration and intestinal infusion, levodopa pharmacokinetics has previously been described both with one- and two-compartment models^10,17^. The data following continuous infusion did in our case not allow for an estimation of a second, peripheral compartment. The estimated typical value for levodopa CL/F following treatment with LCIG was 28 L/h/70 kg (95% SIR CI 25–32 L/h). This is in agreement with previous reported values, from population pharmacokinetic studies that included advanced PD patients that received high doses co-administered with carbidopa. Othman et al.^10^ reported a CL/F of 25 (95% CI 20–27) L/h for levodopa administered as intestinal infusion, Jorga et al.^18^ reported separate levodopa CL/F for a fluctuating and non-fluctuating patient population, of 25 L/h and 29 L/h respectively, and Simon et al.^17^ reported a CL/F of 37 (95% CI 31–43) L/h for oral levodopa/carbidopa administration. The previously reported values for V/F vary widely, between 43 and 131 L^10,17,18^. We estimated V/F to 75 L/70 kg (95% SIR CI 63–88 L), which is in line with the previously reported estimates. The wide difference in estimates could be a result from differences in the study population (e.g. disease severity), the doses administered, blood sampling time points, as well as the route of administration. As an example, Jorga et al.^18^ estimated different V/F, for the fluctuating and non-fluctuating patient population (99 and 124 L respectively).

The CL/F with entacapone addition was estimated be 37% lower (95% SIR CI 33–39%), i.e. 17.7 L/h/70 kg, compared with LCIG. In the previous non-compartmental analysis, it was observed that the plasma concentration was increasing over time with LECIG, and that the doses had not been adjusted appropriately with the addition of entacapone. The current analysis, using population modelling, has the advantage that extra doses and oral dosing are appropriately taken into account, and that the change in plasma concentration over time can be described. Another advantage is that the variability between individuals, as well as the magnitude of the unexplained variability, can be handled with a model based analysis. The conclusion from this analysis is that the continuous maintenance dose should be reduced by approximately 35%, on a population level, when entacapone is simultaneously infused. This is in contrast to the previously suggested reduction by 20% when an LCIG infusion is administered with oral entacapone^4^. Entacapone undergoes extensive first-pass metabolism. A recently developed model, investigating entacapone pharmacokinetics suggested that 6–11% is lost due to intestinal metabolism^19^. The immediate delivery of entacapone to the small intestine with the infusion, and perhaps a shorter intestinal residence time, may result in a higher bioavailability of entacapone, and thereby higher inhibition of COMT compared with oral administration. An infusion of entacapone results in an even plasma concentration, as opposed to oral administration, where administration every 5 h could result in decreased inhibition before next dose intake and more fluctuations in levodopa plasma concentration, although this was not observed in the infusion study with oral entacapone administration^4^. Maximum inhibition of COMT is probably reached early on during the infusion since entacapone reaches steady state within 1 h and because previous studies indicate that there is no delay between maximum entacapone plasma concentration and COMT inhibition^19^.

From the observed plasma concentration–time curve, there is a tendency that the model initially over predicts the plasma concentration following LECIG administration (Fig. 1). The reason for this low initial levodopa concentration is not clear, but has been observed previously with oral multiple-dose administration of levodopa/entacapone/carbidopa^20,21^. One suggested reason for the observed slower absorption was that more levodopa was available with the addition of COMT inhibitor, and that is thereby competing with itself for the saturable large neutral amino acid transporters that transport levodopa across the intestinal membrane. It was also suggested that the delay in absorption could be related to a delayed gastric emptying, caused by higher levodopa concentrations, however this would not be an influencing factor in this study with the infusion treatment, which is bypassing the gastric emptying. Entacapone has molecular similarities to levodopa, and may also compete for transport across the intestinal membrane with levodopa, potentially affecting the rate of levodopa absorption, however, this has been investigated for one of the transporters responsible for levodopa transport and not been found to be the case^22^.

The data did not allow for an estimation of a difference in rate of absorption or volume of distribution between the investigated treatments and thus it is difficult to make conclusions regarding any adjustments of the morning bolus. To investigate this further, administration of bolus doses only, and/or repeated sampling at a longer time period post dosing could be informative.

A formal covariate analysis of the effect of genotype on CL/F was not performed, due to the low number of included subjects, and with relatively high shrinkage in CL/F shift parameter (23.5%), the results are primarily exploratory. The comparison in CL/F based on the different genotypes was only graphically investigated. Corvol et al.^5^ found a significant decrease in CL/F for both low (by 25%) and high (by 40%) activity COMT groups (according to COMT_SNP_ rs4680) when oral levodopa/carbidopa was co-administered with 200 mg of oral entacapone. The decrease in the group with high COMT activity was significantly higher compared to the low activity group. Similarly, we found that CL/F decreased for all individuals (95% SIR CI 34–40%), regardless of genotype, with the addition of entacapone. In contrast, we do not see any clear trend in the decrease in CL/F for patients with high COMT activity compared to other COMT_SNP_ subgroups. The results suggest that all patients, irrespective of COMT rs4680 polymorphism, have a high reduction in CL/F with an addition of simultaneously infused entacapone. No clear trend was observed between administered doses of entacapone and the model predicted decrease in CL/F (data not shown), so a dose dependent decrease in CL/F was not explored.

The plasma concentrations were variable within an individual, with trends observed around the time points of food intake. Protein intake was therefore investigated as a covariate on the rate of absorption and relative bioavailability. Protein intake may interact on transporters in the gastro-intestinal tract and across the blood–brain barrier, possibly causing lower levodopa plasma concentration and an absence or delay of effect after dose intake in patients^23^. However, possibly due to high inter-individual variability and few individuals, it was not possible to characterize the food effect in the present model. Further, to study the food effect was not one of the objectives of the study, and the sampling times were not optimized for this investigation. The variability in plasma concentration over time observed in the data could also be due to other effects, such as differences in gastro-intestinal motility, and overall mobility of the patients, which could coincide with food intake.

## Conclusion

The CL/F is estimated to be 36.5% lower with simultaneous infusion of entacapone. When switching from LCIG to LECIG, our results suggest that the continuous maintenance dose needs to be decreased by approximately 35% on a population level. An effect from entacapone was identified on all individuals, regardless of COMT_SNP_ polymorphism.

## Data Availability

The data that support the findings of this study are available from Lobsor Pharmaceuticals AB. Restrictions apply to the availability of these data, which were used under license for this study. Data are available from the authors with the permission of Lobsor Pharmaceuticals AB.
